# Do Peer Navigators Improve Initiation and Retention in HIV/VH/STI Treatment Programs for People From Key Populations? A Systematic Review of Effectiveness, Values and Preferences, and Cost

**DOI:** 10.1097/QAI.0000000000003364

**Published:** 2024-02-02

**Authors:** Caitlin E. Kennedy, Ping T. Yeh, Annette Verster, Niklas Luhmann, Van T. T. Nguyen, Maeve B. de Mello, Rachel Baggaley, Virginia Macdonald

**Affiliations:** a Department of International Health, Johns Hopkins Bloomberg School of Public Health, Baltimore, MD; and; b Department of Global HIV, Hepatitis and STI Programmes, World Health Organization, Geneva, Switzerland.

**Keywords:** peer navigators, HIV, viral hepatitis, sexually transmitted infections, systematic review, key populations

## Abstract

**Background:**

Key populations are disproportionately affected by HIV, viral hepatitis (VH), and sexually transmitted infections (STIs) and face barriers to care. Peer navigation programs are widely used, but evidence supporting their use has not been synthesized.

**Setting:**

Peer navigation programs for sex workers, men who have sex with men, people who inject drugs, prisoners, and trans and gender diverse people globally.

**Methods:**

To inform World Health Organization guidelines, we conducted a systematic review of effectiveness, values and preferences, and cost studies published between January 2010 and May 2021. We searched CINAHL, PsycINFO, PubMed, and EMBASE; screened abstracts; and extracted data in duplicate. The effectiveness review included randomized controlled trials and comparative observational studies evaluating time to diagnosis or linkage to care, treatment initiation, treatment retention/completion, viral load, cure, or mortality. We assessed risk of bias and summarized findings in GRADE evidence profiles. Values and preferences and cost data were summarized descriptively.

**Results:**

Four studies evaluated the effectiveness of peer navigators for key populations. All were focused on HIV; none were designed for VH or STIs. These studies showed mixed effects on linkage to care, treatment retention/completion, and viral load; no studies measured treatment initiation, cure, or mortality. Two values and preferences studies with community-based organization staff and health workers suggested peer navigators for key populations were acceptable and valued, although continued challenges remained. No cost studies were identified.

**Conclusions:**

Although limited, available studies provide moderate certainty evidence for benefits of HIV/VH/STI peer navigation programs for key populations. Further evaluations are needed.

## INTRODUCTION

Key populations—sex workers, men who have sex with men, people who inject drugs, prisoners, and trans and gender diverse individuals—are disproportionately affected by HIV, viral hepatitis (VH), and sexually transmitted infections (STIs) and face substantial barriers to engagement in care.

Peer navigation programs may be particularly helpful for improving access and outcomes for key populations and are widely used.^1^ Peer navigation is rooted in the concept of *patient navigation*, where vulnerable patients are directly assisted to help find their way through complex health care systems to obtain timely diagnosis and treatment.^2^ Instead of formal health workers, lay staff members who could be considered peers of the participants and could promote trust among the population fill this role.^3^ Peer navigators are often employed at community-based services, primary health care settings, and testing and treatment facilities that are designed to serve people from key population groups. Their role is to support individuals after a reactive screening test to access confirmatory diagnosis, treatment services and to support the early stage of treatment with regular peer support and accompanying individuals to appointments and supporting navigation to other related health services, although the exact role of peer navigators can differ across programs.^4^

Previous randomized controlled trials (RCTs) among general populations have indicated positive effects of peer navigation for HIV/VH/STIs, but these findings are not consistent across all outcomes. For example, HIV peer navigators in the United States were shown to result in improved retention in HIV care, but did not show significant effects on viral suppression at 12 months.^5^ For hepatitis C virus (HCV), a trial of peer navigators in the United Kingdom found improved engagement in care.^6^

Before 2022, the World Health Organization (WHO) had no recommendations specifically on peer navigation for HIV/VH/STI services among key population. However, they did provide several related recommendations. The 2017 WHO Guidelines on Hepatitis B and C testing gave a conditional recommendation that evidence-based interventions like “peer and lay health worker support in community-based settings (moderate certainty) […] should be considered to promote uptake of hepatitis testing and linkage to care and treatment initiation”.^7^ The 2021 WHO Consolidated Guidelines on HIV prevention, testing, treatment, service delivery, and monitoring gave a strong recommendation that “[f]ollowing an HIV diagnosis, a package of support interventions should be offered to ensure timely linkage to care for all people living with HIV” including such interventions as “peer support and navigation approaches for linkage” where peer support included peer counseling.^8^

To inform recently updated WHO HIV/VH/STI guidelines for key populations,^9^ we conducted a systematic review of the evidence for peer navigation programs. Following the WHO guideline development process,^10^ this review evaluated the evidence in 3 related areas: the effectiveness of peer navigation programs, the values and preferences of clients and health workers related to these programs, and the costs or cost-effectiveness of these programs.

## METHODS

### Effectiveness Review

The effectiveness review was designed according to the PICO form as follows: Do peer navigators improve initiation and retention in HIV/VH/STI treatment programs for people from key populations?

- **P**opulations: Key populations as identified by WHO: men who have sex with men, sex workers, people who inject drugs, trans and gender diverse people, and prisoners or people in closed settings
- **I**nterventions: Peer navigation for HIV/VH/STI treatment programs. We focused only on treatment programs, and therefore did not include interventions using peer navigators for HIV pre-exposure prophylaxis, peer navigators for HIV testing, peer education, or peer referral programs
- **C**omparator: No peer navigation
- **O**utcomes:
  - Time to diagnosis (time between initial reactive test result and confirmatory diagnosis) or linkage to care (time between initial reactive test result and engagement in HIV/VH/STI care and treatment services)
  - Treatment initiation for HIV/VH/STIs
  - Treatment retention/completion for HIV/VH/STIs
  - Viral load (eg, HIV and HCV)
  - Cure (for HCV and bacterial STIs, eg, syphilis, gonorrhoea)
  - Mortality

Inclusion criteria for this review were as follows:

- Study design: RCTs or observational studies that compared the intervention vs. the comparison
- Measured 1 or more of the outcomes of interest
- Published in a peer-reviewed journal from January 1, 2010 through the search date of May 27, 2021

No restrictions were placed based on location of the intervention or language of the publication.

We searched 4 databases (CINAHL, PsycINFO, PubMed, and EMBASE) for relevant peer-reviewed publications. Search terms covered concepts for key populations, infections (HIV, VH, and STIs), and peer navigator interventions. The full search strategy for PubMed is available in Text, Supplementary Digital Content, http://links.lww.com/QAI/C193; this was adapted to other databases. This search was complemented by several other ways of identifying articles. First, we ran 2 earlier searches for behavioural interventions for key populations more broadly—1 in 2019 as part of an original scoping review and 1 in March 2020 as part of a search just for RCTs. Articles identified through these previous searches were included in the review. Second, we hand-searched the references of articles identified for inclusion in the review. Third, we contacted experts in the field (including guideline development group members) to identify any additional articles we may have missed.

Titles, abstracts, citation information, and descriptor terms of citations identified through the search strategy were screened for initial inclusion. Full-text articles were obtained of all selected abstracts, and 2 independent reviewers assessed all full-text articles for eligibility to determine final study selection. Differences were resolved through consensus. Data were extracted independently by 2 reviewers using standardized data extraction forms in Excel. Differences in data extraction were resolved through consensus and referral to a senior study team member when necessary.

The following information was gathered from each included study in the effectiveness review:

- Citation information (author, year, title, journal, and language of article)
- Location (country, urban/rural, World Bank income classification, and WHO region)
- Key population (men who have sex with men, sex workers, people who inject drugs, trans and gender diverse people, and prisoners or people in closed settings) and description (gender, age, and specific characteristics reported by the article)
- Sample size (n)
- Study design (specific study design; RCT vs observational; follow-up periods and loss to follow-up)
- Intervention summary and longer description (including who delivered intervention, where intervention was provided, how long/frequent intervention was)
- Comparator description
- Study outcomes (based on PICO above)
- Study outcomes (analytic approach, outcome measures/definitions, intervention vs comparison group, n/%, or effect sizes with confidence intervals or significance levels, conclusions, and limitations)

For randomized trials, risk of bias was assessed using the Cochrane Collaboration's tool for assessing risk of bias.^11^ Methodological components of the studies were assessed and classified as high or low risk of bias. For studies that are not randomized trials but were comparative, study rigor was assessed using the ROBINS-I tool.^12^

Data were analyzed according to coding categories and outcomes. Where there were multiple studies reporting the same outcome for the same intervention–comparator comparison for the same population, we planned to conduct meta-analysis using random effects models. All outcomes were stratified and presented by the 5 specific key populations while recognizing intersectionality across these groups. Findings were summarized in GRADE Evidence Profile tables using GRADEPro. Reporting followed PRISMA guidelines.^13^

### Values and Preferences Review

The same search terms were used to search and screen for studies to be included in the values and preferences review. Studies were included in this review if they presented primary data examining the values and preferences of potential beneficiaries, communities, providers, and stakeholders in relation to peer navigator interventions. We were interested in studies that reported on how potential users felt about peer navigation programs, and how they aligned with their values and preferences around what they would want to experience with such programs. These studies could use qualitative or quantitative methods, but had to present primary data collection—think pieces and review articles were not included. Values and preferences literature was summarized qualitatively and was organized by study design and methodology, location, and population.

### Cost and Resource Needs

The same search terms were used to search and screen for studies to be included in the cost review. Studies were included in this review if they presented primary data comparing costing, unit costs, cost-effectiveness, cost-utility, or cost-benefit of the intervention and comparison listed in the PICO question above or if they presented cost-effectiveness of the intervention as it relates to the PICO outcomes listed above. We planned to summarize cost literature summarized qualitatively. We planned to organize cost literature into 4 categories (health sector costs, other sector costs, patient/family costs, and productivity impacts) and within each category present it by study design/methodology, location, and population.

Findings are presented separated by effectiveness, values and preferences, and cost reviews.

### Role of Funding Source

The funder of the study had no role in study design, data collection, data analysis, data interpretation, or writing of the report.

## RESULTS

**FIGURE 1.**
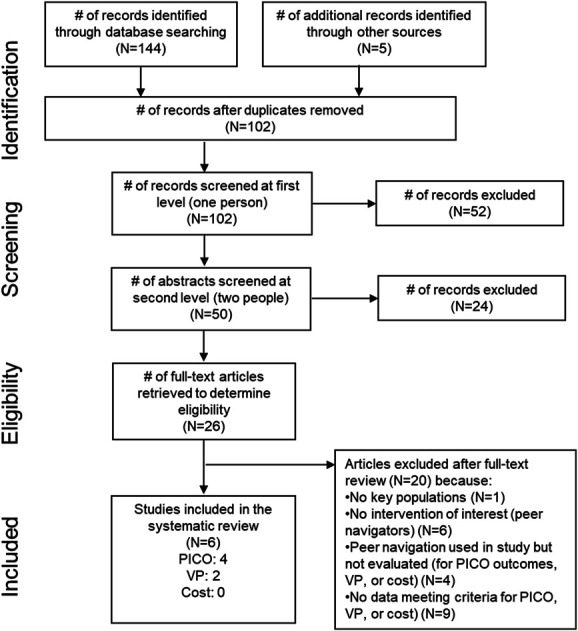
Flow chart showing inclusion of articles across the stages of the systematic review.

Figure 1 presents the flow chart showing inclusion of articles in the systematic review. We identified 4 articles meeting the inclusion criteria for the effectiveness review, 2 for values and preferences, and 0 for costs.

Overall, 4 studies met the inclusion criteria for the effectiveness review.^3,14–16^ Table 1 provides a description of studies included in the effectiveness review. All 4 studies focused on HIV. There were 2 RCTs^3,14^ and 2 observational studies^15,16^; of the observational studies, 1 was a before/after study^15^ and 1 was a cross-sectional dose–response analysis of a single-arm study.^16^ Two studies were conducted in the United States, 1 with transgender women of color^16^ and 1 with prisoners, of which 15% were transgender individuals.^3^ The study with transgender women of color included contingency management (CM) (payment for achievement of target behaviors/goals) and peer navigation.^16^ The study with prisoners focused on peer navigation for transition out of prison.^3^ The other 2 studies were conducted in with sex workers in the Dominican Republic^14^ and Tanzania.^15^ These both evaluated multicomponent, community empowerment interventions with sex workers that also included a peer navigation component.^14,15^

**TABLE 1.** Description of Studies Included in the Effectiveness Review

Study	Location	Study Design	Key Population	Disease Focus	Intervention	Comparison	Outcomes
Cunningham et al^3^	United States	RCT	Prisoners (15% transgender women)	HIV	Peer navigation for transition out of prison	Standard of care	Time to linkage Retention Viral load
Kerrigan et al^14^	Dominican Republic	Before/after	Sex workers	HIV	Multicomponent intervention, including peer navigators to ensure access to and retention in HIV care services and social support	Standard of care	Retention Viral load
Kerrigan et al^15^	Tanzania	RCT	Sex workers	HIV	Multicomponent intervention, including peer navigators to navigate HIV care services	Standard of care	Time to linkage Retention Viral load
Reback et al^16^	United States	Cross-sectional dose–response analysis of a one-arm study	Transgender women	HIV	Peer navigation and CM (more sessions attended)	Fewer sessions attended	Retention Viral load

Table 1, Supplemental Digital Content, http://links.lww.com/QAI/C193, presents the risk of bias assessments for these studies. The 2 RCTs were rated low for overall risk of bias. Although the RCT with prisoners had high loss to follow-up, it was similar in both arms.^3^ The RCT with sex workers was rated low risk of bias overall, but moderate in measurement of the outcome as some outcomes were self-report.^15^ Similarly, both observational studies had low risk of bias overall. The before/after study with sex workers was rated moderate for risk of bias in measurement of the outcome as some outcomes were self-report.^14^ Finally, the study with transgender women of color was rated as high risk of bias due to missing data because there was high attrition in the study over time.^16^

Table 2, Supplemental Digital Content, http://links.lww.com/QAI/C193, presents the GRADE evidence profile for all outcomes. The RCTs provided data for 3 of our PICO outcomes: (1) time to diagnosis or linkage to care; (2) treatment retention/completion for HIV/VH/STIs; and (4) viral load (eg, HIV and HCV). The observational studies measured the same outcomes. No studies measured our other outcomes of interest: treatment initiation for HIV/VH/STIs, cure for curable STIs, or mortality. We did not conduct meta-analysis because of lack of data from multiple studies on the same measured outcome and the diversity of studies in terms of interventions, populations, and study designs.

Three studies measured time to diagnosis or linkage to care. One RCT with high certainty showed peer navigators made no difference in probability of HIV care visits after jail release among prisoners in the United States.^3^ A low certainty RCT among sex workers in Tanzania showed peer navigators improved ever linkage to HIV care.^15^ The moderate certainty observational study of CM and peer educators showed improvements in transgender women of color in the United States having a first HIV care visit.^16^

For treatment retention/completion, all 3 studies measuring this outcome reported current use of antiretroviral treatment (ART). The RCT among prisoners in the United States showed no difference in ART use with peer navigators (high certainty),^3^ whereas both the RCT and before/after study with sex workers showed modest improvements in ART use (low certainty).^14,15^

All 4 studies measured viral load (high to moderate certainty). However, none showed a substantial impact of peer navigators on viral load except for the observational study using CM with transgender individuals in the United States.^16^

Overall, 2 studies were included in the values and preferences review (Table 2).^17,18^ Both were qualitative studies conducted with health care providers or staff at community-based organizations (CBOs) serving key populations; no studies were conducted with key populations directly. Of the 2 studies identified, 1 was conducted in Kenya^17^ and 1 in Mexico.^18^ Table 2 provides descriptive data for these studies and key findings from each. In Kenya, previously successful “mother mentor” programs instilled confidence in the similar success of peer navigator programs for men who have sex with men, and overloaded health workers welcomed assistance from peer navigators and suggested appropriate tasks for them.^17^ However, concerns remained about clients becoming too dependent on peer navigators (not able to self-manage) and community reactions to support groups for men who have sex with men. In Mexico, CBO staff saw a continued need to address social factors such as culturally competent care, stigma, lack of social support, and care disengagement.^18^ They felt peer navigators could address stigma and social support. However, they discussed concerns about peer navigators managing police interference.

**TABLE 2.** Descriptions of Values and Preferences Studies

Study	Location, Population Description	Study Design: Methods, Sample Size	Key Values and Preferences Findings
Micheni et al,^17^	Kenya: Mombasa and Kilifi, health care workers serving men who have sex with men	Qualitative: focus groups, 4 groups with 29 total participants	1. The successful peer mentor programs, particularly the ‘‘mother mentor’’ program for prevention of mother to child transmission, seemed to instill confidence in the feasibility and acceptability of advancing similar programs for men who have sex with men. 2. Health care workers welcomed the idea of help from peer navigators, citing the heavy clinic workloads and time constraints typical of most public health facilities. They proposed that peers help provide psychosocial support and health information, and remind patients of medication-taking and clinic visits. Health care workers, however, emphasized the need for patients receiving peer support to maintain regular contact with health care workers at clinics. 3. Enthusiasm for using peer navigators to provide adherence support for men who have sex with men was tempered by concerns that patients ultimately need to become self-sufficient and take medications independently after a limited period of support rather than become dependent on peer navigators for continued assistance 4. Community reactions to peer support programs for men who have sex with men were also of concern because of perceptions among community members that men who have sex with men receive additional services that are not available to others. Some health care workers felt that such interventions could consequently jeopardize safety for the community of men who have sex with men, whereas others saw this as an opportunity for sensitization and dialogue.
Pitpitan et al,^18^	Mexico: Tijuana, CBO workers serving key populations (men who have sex with men, trans and gender diverse people, and people who inject drugs)	Qualitative: in-depth interview and open-ended survey, 12 participants	1. Concerns were raised about how a peer navigator program would manage potential police interference (eg, confiscation or disposal of ART by police who assume they are illicit drugs, clients having to cross through areas in Zona Norte where police set up raids or check-points to access HIV care, prevention, or drug treatment; the potential for the peer navigators to draw police attention to their clients or for the peer navigators themselves to be perceived by the police to be people who inject drugs or female sex workers and be harassed vis-a-vis street outreach activities). 2. Participants described challenges in patient tracking and monitoring for highly mobile key populations. The key informants described how many patients become lost to follow-up because clients frequently relocated within the city or to other jurisdictions and how the national HIV surveillance system did not allow for identifying or re-engaging those lost to follow-up (unsubscribed patient if missed too many appointments or go too long without an appointment) so depending on individual staff at CBOs to pull together resources and coordinate efforts to locate these patients. 3. Key informants also emphasized the need to address a range of social factors affecting the target populations, namely culturally competent care, stigma, and lack of social support 4. Key informants discussed how despite the availability of telemedicine at the community-based organization, there were still barriers to linkage and retention to HIV care that could be addressed. Specifically, by having peer navigators work with patients, it was perceived that the program could help to reduce stigma and provide a source of social support.

No included studies presented primary data examining cost-effectiveness, cost-utility, or cost-benefit for peer navigators for key populations.

## DISCUSSION

This systematic review identified 4 studies evaluating the effectiveness of peer navigators for key populations on our outcomes of interest. These 4 studies were conducted with a range of key populations, although none included people who use drugs or men who have sex with men. All focused on peer navigation for HIV; none were designed for VH or STIs. They provided high-to-moderate certainty evidence on most of our outcomes of interest, although no studies measured treatment initiation, cure, or mortality. Overall, the evidence for outcomes of linkage to care, treatment retention/completion, and viral load was mixed. Even the highest certainty evidence, from a study among prisoners in the United States, found peer navigation led to no difference in probability of HIV care visits after jail release but showed improvements in undetectable viral load.^3^ The mixed effects seen across and within studies are difficult to explain with certainty but may be because of differences in study populations, locations, study designs (such as selection of follow-up times, comparison groups, or specific outcome measures), or interventions. Importantly, all 4 interventions were different, and most included other critical intervention features such as community empowerment or contingency management in addition to peer navigation, making it difficult to tease out the unique effects of peer navigation.

Our review also identified 2 values and preferences studies, which found that CBO workers and health workers valued peer navigation programs for key populations, although continued programmatic challenges remained. Stigma, criminalization, and negative community attitudes toward key populations remained key barriers to programmatic success, although peer navigation programs were also seen as helping to address issues of stigma. Unfortunately, we identified no studies that assessed the values and preferences of individuals from key populations directly and how they felt about HIV/VH/STI peer navigation programs. However, the WHO guideline development process did include work led by key population networks to assess values and preferences, including of peer navigation.^8^ They found that the services provided by peer navigators were highly valued, along with their capacity to “act as a bridge between 2 different worlds” and reach community members.

We also identified no studies for the cost review. However, we may be able to estimate costs from other peer navigation programs not designed for key populations.^19^ Full program costs, cost-effectiveness, and affordability will vary across settings by the specific tasks peer navigators are asked to complete in different programs. Resource requirements needed may include cost for full-time salaries of peer navigators, cost for additional supervisory staff/staff time, potential costs to contract with government or other facilities, and both initial and recurring costs, including mobile phones, SIM cards/credit, and transportation.^1^ In some settings, peer navigators may be paid less than other health professionals to perform the same tasks, although it is important that they are adequately renumerated and afforded the same employment conditions as other members of staff. However, if peer navigation is added on top of existing services, there may be an increase in total program costs.

Although our included studies were not sufficient to conduct a rigorous assessment of the impact of peer navigators on health equity, there is potential for peer navigation to help support the most vulnerable populations and increase their health care accessibility. Peer navigators may make clients feel more comfortable with services that meet their specific needs and serve as role models, increasing equity. Peer navigators may be more able to build trust with clients,^4^ which may be particularly important for key populations, who often experience social marginalization and criminalization, and stigma from health care services.^20^ As shown in studies of peer navigators for HIV prevention services, peer navigators can provide psychosocial support to help clients who are anxious about their test results or hesitant to go for confirmatory testing and treatment. There may also be a positive equity impact of such programs through empowerment of peer navigators themselves. Conversely, there may be a negative equity impact if programs do not pay peer navigators or value them less than other members of the care team.

The evidence from this review was used to inform the 2022 WHO consolidated guidelines on HIV, VH and STI prevention, diagnosis, treatment, and care for key populations.^9^ Per the WHO guideline development process,^10^ evidence on effectiveness, values and preferences, and cost from this review was used in combination with additional considerations of equity and acceptability and input from key populations and other stakeholders, including commissioned qualitative research from global networks of key populations on values and preferences among their networks. After deliberation, the guideline development group agreed to develop a new conditional recommendation on peer navigation, based on an overall moderate certainty of evidence. The specific guideline statement and remarks included in the WHO guidelines^9^ read as follows:

- “Recommendation:
- Peer navigators are recommended to support people from key populations to start HIV, viral hepatitis or STI treatment, and to remain in care (conditional recommendation, moderate certainty of evidence).
- Remarks:
- A peer navigator's role is to assist key population members to access health services, navigate these services and stay in care.
- Peer navigators require adequate remuneration, recognition, training and other support to fulfil their role.
- Peer navigators are often highly valued by their peers.”

Strengths of our review include our broad focus on all key populations and regions, rigorous search and synthesis of evidence for the effectiveness review, and collection of complementary data on values and preferences and cost. Although we conducted a thorough search using multiple databases and search strategies, we only included peer-reviewed articles and may have missed some valuable information from the grey literature, particularly for the values and preferences and cost reviews. Several included studies presented multicomponent interventions, so we were not able to isolate the effects of the peer navigation component and had to downgrade these studies for indirectness in the GRADE system. In addition, our inclusion criteria focused on peer navigation programs that were specifically designed to serve key populations. We excluded evaluations of several programs that were not limited to key populations, but where a substantial portion of their clients were from key populations; for example, 1 study of an HCV peer navigation program recruited people with a lifetime history of injection drug use, but only two-thirds of participants were currently injecting drugs (last 30 days); this trial found that peer navigators successfully reached marginalized individuals and increased HCV knowledge but did not improve linkage to HCV care after diagnosis.^21^ In many cases, peer navigation programs identified peers as other people living with HIV/VH/STIs rather than seeing peers necessarily as other members of a key population group. Further research could examine these types of peer navigation programs and how well they work for key populations.

Ultimately, our conclusions from this review are also limited by the availability of the existing literature. Although our included studies for the effectiveness review were diverse in terms of populations and locations, there were only 4. There was even more limited data on values and preferences, nothing on cost, and no studies looking at peer navigation programs for VH or STIs. Future research would be useful to expand this evidence base across a range of settings with consideration of the diverse needs of each of the key population groups and unique treatment characteristics of the different diseases. However, our overall conclusions in support of peer navigation programs are strengthened by the positive experiences and perspectives on peer navigation programs for key populations that have focused on pre-exposure prophylaxis or HIV testing, instead of treatment support.^22–24^

Although limited, available studies provide moderate certainty evidence for the potential benefits of HIV/VH/STI peer navigation programs for key populations. Further evaluations of programs designed for key populations are needed.

## Supplementary Material
