# Supplementary material for: Do Peer Navigators Improve Initiation and Retention in HIV/VH/STI Treatment Programs for People From Key Populations? A Systematic Review of Effectiveness, Values and Preferences, and Cost
Source: J Acquir Immune Defic Syndr. 2024 Feb 2;95(4):305–12. doi: 10.1097/QAI.0000000000003364 (PMC10896188; doi:10.1097/QAI.0000000000003364)
Supplement: Supplementary file 1 [file qai-95-305-s001.docx]

**Do peer navigators improve initiation and retention in HIV/VH/STI treatment programmes for people from key populations?** **A systematic review of effectiveness, values and preferences, and cost**

**Text, Supplementary Digital Content 1. Search Strategy for Pubmed.**

**Concept 1: Key populations**

Concept 1a: Sex workers

(Sex Workers [MeSH] OR Sex work [MeSH] OR sex work [tw] OR prostitut* [tw] OR commercial sex [tw] OR transactional sex [tw] OR SW [tw] OR FSW [tw] OR CSW [tw] OR sex trade [tw] OR trade sex [tw] OR sex industry [tw] OR entertainment worker [tw])

OR

Concept 1b: Men who have sex with men

(Homosexuality, Male [Mesh] OR gay men [tw] OR gay man [tw] OR gay male [tw] OR homosexual [tw] OR MSM [tw] OR men who have sex with men [tw] OR males who have sex with males [tw] OR bisexual men [tw] OR bisexual man [tw] OR bisexual male [tw] OR gay and bisexual men [tw] OR gay and other men who have sex with men [tw])

OR

Concept 1c: People who inject drugs

(Drug Users [Mesh] OR Substance Abuse, Intravenous [Mesh] OR drug use [tw] OR drug user [tw] OR drug users [tw] OR intravenous drug user [tw] OR injecting drug user [tw] OR injection drug user [tw] OR drug abuse* [tw] OR substance use [tw] OR substance abuse* [tw] OR people who inject drugs [tw] OR people who use drugs [tw] OR IVDU [tw] OR IDU [tw] OR PWUD [tw] OR PWID [tw] OR drug usage [tw])

OR

Concept 1d: Trans and gender diverse people

(Transgender Persons [Mesh] OR transgender [tw] OR transsexual [tw] OR transvest* [tw] OR travesti* [tw] OR trans [tw] OR koti [tw] OR hijra [tw] OR mahu [tw] OR waria [tw] OR katoey [tw] OR berdache [tw] OR muxe [tw] OR third spirit [tw] OR third spirited [tw] OR assigned male at birth [tw] OR assigned female at birth [tw] OR AMAB [tw] OR AFAB [tw] OR takataapui [tw] OR tahine [tw] OR whakawahine [tw] OR tangata ira wahine [tw] OR tangata ira tane [tw] OR irawhiti [tw] OR irahuhua [tw] OR MTF [tw] OR FTM [tw] OR transmasculine [tw] OR trans masculine [tw] OR transfeminine [tw] OR trans feminine [tw] OR people of trans experience [tw] OR non-binary [tw] OR nonbinary [tw] OR gender non-conforming [tw] OR genderqueer [tw] OR gender diverse [tw])

OR

Concept 1e: Prisoners and people in closed settings

(Prison [Mesh] OR Prisoners [Mesh] OR Criminals [Mesh] OR Concentration camps [Mesh] OR incarcerat* [tw] OR prison* [tw] OR jail* [tw] OR penitentiary [tw] OR penitentiaries [tw] OR penal institution [tw] OR correctional center [tw] OR correctional centre [tw] OR correctional facility [tw] OR correctional facilities [tw] OR correctional setting [tw] OR detain* [tw] OR detention center [tw] OR detention centre [tw] OR inmate [tw] OR imprison* [tw])

OR

Concept 1f: general key pops terms

(key population [tw] OR most at risk population [tw] OR MARPS [tw] OR vulnerable population [tw])

**AND**

**Concept 2: HIV, Viral Hepatitis, STIs**

(HIV [Mesh] OR Acquired Immunodeficiency Syndrome [Mesh] OR HIV Infections [Mesh] OR human immunodeficiency virus [tiab] OR acquired immunodeficiency syndrome [tiab] OR HIV [tiab] OR AIDS [tiab] OR HIV1 [tiab] OR HIV2 [tiab] or Hepatitis, Chronic [Mesh] OR Hepatitis, Viral, Human [Mesh] OR hepatitis b [tiab] OR HBV [tiab] OR hepatitis c [tiab] OR HCV [tiab] OR hepatitis d [tiab] OR HDV [tiab] Or Sexually transmitted diseases [Mesh] OR STI [tiab] OR STD [tiab] OR sexually transmitted infection [tiab] OR sexually transmitted disease [tiab] OR sexually transmitted disorder [tiab] OR sexually transmissible infection [tiab] OR sexually transmissible disease [tiab] OR sexually transmissible disorder [tiab] OR anogenital wart [tiab] OR bacterial vaginosis [tiab] OR candida albicans [tiab] OR candidal vaginitis [tiab] OR candidiasis[tiab] OR candidosis [tiab] OR chancroid [tiab] OR chlamydia [tiab] OR Condylomata Acuminata [tiab] OR donovanosis [tiab] OR genital disorder [tiab] OR Gardnerella [tiab] OR genital infection [tiab] OR genital ulcer [tiab] OR genital wart [tiab] OR gonorrhea [tiab] OR gonorrhoea [tiab] OR Neisseria gonorrhoeae [tiab] OR granuloma inguinale [tiab] OR herpes [tiab] OR HPV [tiab] OR human papillomavirus [tiab] OR monilia albicans [tiab] OR monilial infection [tiab] OR syphilis [tiab] OR Treponema pallidum [tiab] OR trichomonas vaginalis [tiab] OR trichomoniases [tiab] OR trichomoniasis [tiab] OR venereal disease [tiab] OR venereal disorder [tiab] OR vulvitis [tiab] OR vulvovaginitis [tiab])

**AND**

**Concept 3: Peer navigators**

(peer-assisted [tiab] OR peer-led [tiab] OR peer-mentoring [tiab] OR peer education [tiab] OR peer-facilitated [tiab] OR peer intervention [tiab] OR peer health promotion [tiab] OR peer networks [tiab] OR peer-driven [tiab] OR peer-delivered [tiab] OR peer group [tiab] OR peer outreach [tiab] OR care peer [tiab] OR care peers [tiab] OR peer coach [tiab])

AND

(service coordinator [tiab] OR service coordination [tiab] OR service navigator [tiab] OR service navigation [tiab] OR client facilitator [tiab] OR client navigation [tiab] OR client advocate [tiab] OR client advocacy [tiab] OR health coach [tiab] OR patient navigation [tiab] OR patient navigator [tiab] OR patient navigators [tiab] OR guided care [tiab] OR navigator [tiab] OR navigators [tiab] OR post-discharge support [tiab])

**Table, Supplemental Digital Content 2. Risk of bias assessments: Cochrane tool (for RCTs) and ROBINS-I (for observational studies).**

| Cochrane Risk of Bias Tool (for RCTs) | | | | | | | | | | | | | | | |
| --- | --- | --- | --- | --- | --- | --- | --- | --- | --- | --- | --- | --- | --- | --- | --- |
| Risk of bias | | Risk of bias arising from the randomization process | | Risk of bias due to deviations from the intended interventions | | | | Risk of bias due to missing outcome data | | Risk of bias in measurement of the outcome | | Risk of bias in selection of the reported result | | Overall risk of bias judgment | |
| Author Year | |  |  | Effect of assignment to intervention | | Effect of adhering to intervention | |  |  |  |  |  |  |  |  |
| Cunningham et al., 2018 | | Low | | Low | | Low | | Low^1^ | | Low | | Low | | Low | |
| Kerrigan et al., 2019 | | Low | | Low | | Low | | Low | | Moderate^2^ | | Low | | Low | |
| ROBINS-I Tool (for non-RCTs) | | | | | | | | | | | | | | | |
| Author Year | Bias due to confounding | | Bias in selection of participants into the study | | Bias in classification of interventions | | Bias due to deviations from intended intervention | | Bias due to missing data | | Bias in measurement of outcomes | | Bias in selection of the reported result | | Overall risk of bias judgement |
| Kerrigan et al., 2016 | Moderate | | Low | | Low | | Low | | No information | | Moderate^2^ | | Low | | Low risk of bias |
| Reback et al., 2019 | Moderate | | Low | | Low | | Low | | High^3^ | | Low | | Low | | Low risk of bias |

1 Loss to follow-up of approximately 70% in both arms, but similar reasons in both arms.

2 Some outcomes are self-reported

3 High attrition

**Table, Supplemental Digital Content 3. GRADE evidence profile for effectiveness review studies.**

| **Certainty assessment** | | | | | | | **№ of patients** | | **Effect** | | **Certainty** | **Importance** |
| --- | --- | --- | --- | --- | --- | --- | --- | --- | --- | --- | --- | --- |
| **№ of studies** | **Study design** | **Risk of bias** | **Inconsistency** | **Indirectness** | **Imprecision** | **Other considerations** | **peer navigation** | **no peer navigation** | **Relative (95% CI)** | **Absolute (95% CI)** |  |  |
| **Time to diagnosis or linkage to care (follow up: mean 12 months; assessed with: probability of HIV primary care visits after jail release)** | | | | | | | | | | | | |
| 1 ^3,a^ | randomised trials | not serious ^b^ | not serious ^c^ | not serious | not serious | none |  |  | **Probability difference 0.04** (-0.04 to 0.12) |  | ⨁⨁⨁⨁ HIGH ^d,e^ | CRITICAL |
| **Time to diagnosis or linkage to care (follow up: mean 10 months; assessed with: ever linked to HIV care)** | | | | | | | | | | | | |
| 1 ^15,f^ | randomised trials | serious ^g^ | not serious ^c^ | serious ^h^ | not serious | none | 72/91 (79.1%) | 44/80 (55.0%) | **RR 1.44** (1.15 to 1.80) | **242 more per 1,000** (from 82 more to 440 more) | ⨁⨁◯◯ LOW | CRITICAL |
| **Time to diagnosis or linkage to care (assessed with: having a first HIV care visit)^i^** | | | | | | | | | | | | |
| 1 ^14,j^ | observational studies | not serious ^b^ | not serious ^c^ | serious ^k^ | not serious | none |  |  | **Regression coefficient 0.38** (0.09 to 0.67) |  | ⨁⨁⨁◯ MODERATE ^m^ | CRITICAL |
| **Treatment retention/completion for HIV/VH/STIs (follow up: mean 12 months; assessed with: current ART use)** | | | | | | | | | | | | |
| 1 ^3,a^ | randomised trials | serious ^g^ | not serious ^c^ | not serious | not serious | none | 104/125 (83.2%) | 107/125 (85.6%) | **Probability difference -0.01** (-0.06 to 0.03) |  | ⨁⨁⨁◯ MODERATE ^n^ | CRITICAL |
| **Treatment retention/completion for HIV/VH/STIs (follow up: mean 18 months; assessed with: current ART use)** | | | | | | | | | | | | |
| 1 ^15,f^ | randomised trials | serious ^g^ | not serious ^c^ | serious ^h^ | not serious | none | 74/91 (81.3%) | 51/80 (63.7%) | **RR 1.28** (1.05 to 1.55) | **179 more per 1,000** (from 32 more to 351 more) | ⨁⨁◯◯ LOW | CRITICAL |
| **Treatment retention/completion for HIV/VH/STIs (follow up: mean 10 months; assessed with: current ART use)** | | | | | | | | | | | | |
| 1 ^16,o^ | observational studies | serious ^g^ | not serious ^c^ | serious ^p^ | not serious | none | 178/228 (78.1%) | 161/228 (70.6%) | **RR 1.11** (1.03 to 1.19) | **78 more per 1,000** (from 21 more to 134 more) | ⨁⨁◯◯ LOW | CRITICAL |
| **Viral load (follow up: mean 12 months; assessed with: HIV viral suppression: undetectable viral load (<75 copies/mL))** | | | | | | | | | | | | |
| 1 ^3,a^ | randomised trials | not serious ^q^ | not serious ^c^ | not serious | not serious | none | 62/125 (49.6%) | 45/125 (36.0%) | **Probability difference 0.18** (0.02 to 0.40) |  | ⨁⨁⨁⨁ HIGH ^l^ | IMPORTANT |
| **Viral load (follow up: mean 12 months; assessed with: HIV viral suppression (<400 copies/mL))** | | | | | | | | | | | | |
| 1 ^15,f^ | randomised trials | not serious ^q^ | not serious ^c^ | serious ^h^ | not serious | none | 46/91 (50.5%) | 36/76 (47.4%) | **RR 1.07** (0.78 to 1.46) | **33 more per 1,000** (from 104 fewer to 218 more) | ⨁⨁⨁◯ MODERATE | IMPORTANT |
| **Viral load (follow up: mean 10 months; assessed with: HIV detectable viral load (>50 copies/ml))** | | | | | | | | | | | | |
| 1 ^16,o^ | observational studies | not serious ^q^ | not serious ^c^ | serious ^p^ | not serious | none | 116/228 (50.9%) | 114/227 (50.2%) | **RR 1.02** (0.93 to 1.12) | **10 more per 1,000** (from 35 fewer to 60 more) | ⨁⨁⨁◯ MODERATE | IMPORTANT |
| **Viral load (assessed with: HIV undetectable viral load (<20-75 copies/mL))^r^** | | | | | | | | | | | | |
| 1 ^14,j^ | observational studies | not serious ^q^ | not serious ^c^ | serious ^k^ | not serious | none |  |  | **Regression coefficient 0.10** (0.05 to 0.14) |  | ⨁⨁⨁◯ MODERATE | IMPORTANT |

**CI:** Confidence interval; **RR:** Risk ratio

#### Explanations

a. Study description: This RCT among 356 prisoners (including 15% transgender individuals) living with HIV in USA compared peer navigation for transition out of prison to standard of care.

b. Risk of bias: Not downgraded for detection bias. Blinding was not possible given the nature of the intervention. Detection bias was unlikely as participant-reported visit data was validated by comparison with electronic visit records.

c. Inconsistency: This could not be evaluated, as there is only a single study.

d. Additional time points: Linkage to HIV primary care after jail release. 3 month follow-up (n=312): difference: 0.01 (-0.09 to 0.12, p=0.81). 6 month follow-up (n=260): difference: 0.12 (0.04 to 0.22, p=0.01)

e. Number of patients not reported. Effect: probability difference calculated from intervention probability: 0.92 (0.87 to 0.97), control probability: 0.88 (0.82 to 0.94)

f. Study description: This RCT among 171 sex workers living with HIV in Tanzania compared a multi-component intervention, including peer navigators to navigate HIV care services, to standard of care.

g. Risk of bias: Downgraded once for detection bias. Blinding was not possible given the nature of the intervention. Detection bias was possible as data was self-reported and may have been affected by a lack of blinding.

h. Indirectness: Downgraded because intervention was multi-component and included a community-led drop-in center with mobilization activities, sensitivity training for HIV clinical providers and police, venue-based peer-education and condom distribution, and text messages and reminders to promote solidarity and care engagement, in addition to peer navigation.

i. Regression coefficient for estimated associations between attendance to peer navigation sessions and achievement of continency management target (first HIV care visit), using a partial proportional odds ordinal logistic regression.

j. Study description: This cross-sectional dose-response analysis of a one-arm study among 139 transgender and gender diverse people living with HIV in USA provided peer navigation and contingency management (payment for achieving care milestones and compared those who attended fewer sessions to those who attended more sessions.

k. Indirectness: Downgraded because intervention included contingency management in addition to peer navigation.

l. Additional time points: Viral suppression. 3 month follow-up (n=315): difference: 0.16 (0.01 to 0.31, p=0.03).

m. Number of patients not reported. Effect: probability difference calculated from intervention probability: 0.92 (0.87 to 0.97), control probability: 0.88 (0.82 to 0.94)

n. Additional time points: ART use. 3 month follow-up (n=315): difference: -0.01 (-0.06 to 0.04, p=0.69). 6 month follow-up (n=285): difference: 0.02 (-0.05 to 0.08, p=0.65).

o. Study description: This before/after study among 228 sex workers living with HIV in the Dominican Republic compared a multi-component intervention, including peer navigators to ensure access to and retention in HIV care services and social support, to standard of care.

p. Indirectness: Downgraded because intervention was multi-component and included individual counseling and health education by psychologists or social workers, sensitivity training for HIV clinical providers, and community mobilization activities at a sex worker drop-in center, in addition to peer navigation.

q. Risk of bias: Not downgraded for detection bias. Blinding was not possible given the nature of the intervention. Detection bias was unlikely as the outcome was unlikely to have been affected by a lack of blinding.

r. Regression coefficient for estimated associations between attendance to peer navigation sessions and achievement of continency management target (HIV undetectable viral load), using a proportional odds ordinal logistic regression. Positive regression coefficient means attending more sessions was associated with greater achievement of target.
